# Cloning and Phylogenetic Analysis of Sid-1-Like Genes from Aphids

**DOI:** 10.1673/031.008.3001

**Published:** 2008-04-10

**Authors:** Weina Xu, Zhaojun Han

**Affiliations:** Key Laboratory of Monitoring and Management of Plant Diseases and Insects, Ministry of Agriculture, Nanjing Agricultural University, Nanjing 210095, China

**Keywords:** aphid, sid-1 gene, cDNA clone, phylogenetic tree

## Abstract

The sid-1 (systemic interference defective) gene encodes a transmembrane protein that is an important participator in the systemic RNAi pathway and has been reported in several organisms. In insects, sid-1-like genes were described from *Tribolium castaneum*, *Apis mellifera*, *Bombyx mori* and *Schistocerca americana*, but were not found in *Drosophila melanogaster* and *Anopheles gambiae*. To investigate whether this gene occurs in aphid species, RT-PCRs were performed using degenerate primers designed using the conserved motif of sid-1-like genes. An sid-1-like full-length transcript was amplified from the cotton/melon aphid, *Aphis gossypii* Glover (Homopera: Aphididae), and a fragment was amplified from the grain aphid, *Sitobion avenae* (F.). The trancript from *A. gossypii* was 3067 bp long, with an open reading frame encoding 766 amino acids. Sequence analysis indicated that this transcript shares highest similarity with the reported sid-1-like gene in *Schistocerca americana* (53%, fragment), followed by *A. mellifera* (44%), *T. castaneum* (32–44%), *B. mori* (38–42%) and *Caenorhabditis elegans* (25%). Analysis of the transmembrane protein topological structure indicated that the protein encoded by this gene has a similar structure to SID-1 of *C. elegans*. A phylogenetic tree with all available sid-1-like genes suggests that sid-1-like genes may have had a long evolutionary history. Considering its importance in the RNAi pathway, the absence of a sid-1-like gene in *D. melanogaster* and *A. gambiae* is worthy of further investigation.

## Introduction

RNA interference (RNAi) triggered by double strand RNA (dsRNA), which causes selective gene silence by mRNA cleavage or an mRNA translation block, has been widely adopted as powerful tool for functional genomics and holds promise for gene therapeutics. SID-1 protein (systemic interference defective), a recently identified multispan transmembrane protein in the nematode, *Caenorhabditis elegans*, appears to be necessary for systemic RNAi (Winston et al. 2002). This protein consists of 11 transmembrane domains with its N-terminal region outside the cell and the C-terminus inside. It has been shown that SID-1 in *C. elegans* could passively and actively import dsRNA, siRNA or other RNAi signals into different cell lines. Its efficiency is affected by the length of the dsRNA, with a marked preference for being longer than 100 bp (Winston et al. 2002; Feinberg et al. 2003). The sid-1 like gene was shown to be ubiquitous and robustly expressed in cellular membranes of the grasshopper, *Schistocerca americana* (Dong and Friedrich. 2005).

So far, sid-1-like genes have been found in a variety of organisms including microbes, nematodes, insects, fish and mammals, but not in all organisms. Alignment of the *C. elegans* sid-1 amino acid sequence with the genomes of all known organisms on GenBank revealed that sid-1 homologous sequences are present in human, mouse, rat, chimp, cow and dog, but not in sheep, pig and cat (unpublished observations). In insects, it has been reported that *S. americana* (Dong and Friedrich. 2005), *Apis mellifera*, *Bombyx mori* and *Tribolium castaneum* (George and Gene. 2006) had 1–3 sid-1-like transcripts, but they were absent in the genomes of *Drosophila melanogaster* (Roignant et al. 2003) and *Anopheles gambiae* (Blandin et al. 2002, Voinnet. 2005). It is obvious that research on more organisms is needed to determine the distribution and evolution of sid-1-like genes.

Insects with sid-1-like genes, such as *T*. *castaneum* (Tomoyasu and Denell. 2004), show systemic RNAi symptoms, while insects without sid-1-like genes, such as *D*. *melanogaster* (Roignant et al. 2003, Voinnet. 2005) and *A. gambiae* (Blandin et al. 2002, Voinnet. 2005) show only cell-autonomous RNAi. Therefore, to determine whether an insect contains sid-1-like gene is useful for research on RNAi function. In this paper, degenerate PCR is used to clone sid-1-like genes in Aphidoidea.

## Materials and Methods

### Aphid rearing conditions

The cotton/melon aphid, *Aphis gossypii* Glover and the grain aphid, *Sitobion avenae* (F.) (Homoptera: Aphididae) were reared in the laboratory for several generations. The rearing conditions were: 16:8 LD, 25±1°C and 70%–85% relative humidity.

### RNA preparation and cDNA synthesis

Total RNA was extracted from apterous adult aphids using TRIzol reagent (Invitrogen, www.invitrogen.com). Thirty milligrams of aphids were ground in liquid nitrogen in 1.5 ml microtubes and homogenized in 1 mL of TRIzol reagent on ice. The remaining step followed the manufacturer's protocol. First-strand cDNA was synthesized from the total RNA with reverse transcriptase M-MLV and oligod(T)18 (promega, www.promega.com). 5′-and 3′-RACE-ready cDNA were prepared according to the instructions of BD SMART™ RACE cDNA amplification kit's protocol (www.bdbiosciences.com).

### Degenerate PCR amplification

Degenerate primers targeting conserved gene regions were determined by alignment of published sid-1-like transcripts from distantly related species. Primers were synthesized by Invitrogen. Five degenerate primers used were as follows: 
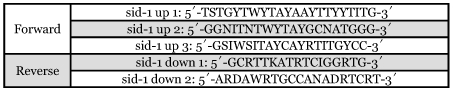
Amplification was performed using 50 µl Takara Taq polymerase system with 8 µM each of the forward and reverse primers and 1 µl cDNA template. Touch down PCR and nest PCR were carried out to amplify the fragments with different primer pairs using a PTC-200 DNA engine thermocycler (MJ Research, www.bio-rad.com). In the successful PCR procedure, the annealing temperature was designed to decrease by 2°/3 cycles from 60° to 50°, and another 20 cycles were added at 50°. PCR products were checked by electrophoresis on 2% w/v agarose gel in TAE buffer and the resulting bands were visualized by ethidium bromide staining. The target product from PCR was isolated on gels of low melting point agarose, purified using Wizard PCR preps DNA purification system (Promega) and then cloned into pGEM-T easy vector (Promega). The ligation reactions were used for transformation of *Escherichia coli* DH5αa competent cells. Successful clones were screened with standard ampicillin selection. Recombinant plasmids were fully sequenced on Applied Biosystems 377 automated sequencer by Invitrogen.

**Figure 1.**
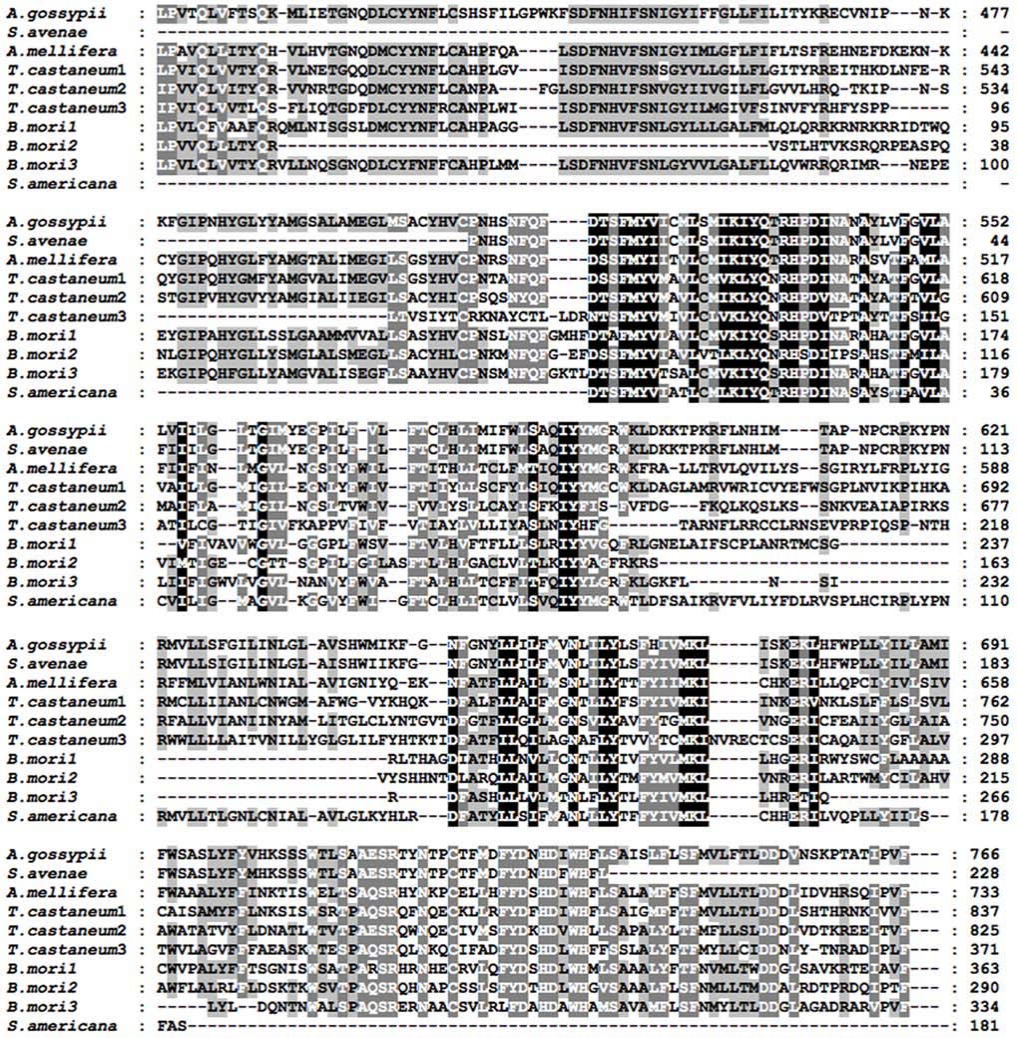
Multiple alignment of amino acid sequences of insect SID-1 proteins (the ends of some genes are not shown in this figure).

### Cloning of the full length from *Aphis gossypii* with RACE strategy

According to the sid-1-like transcript fragment amplified from cotton aphid by degenerate primers, specific primers were designed and used to amplify the full length. Primers are as follows: 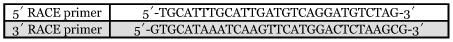
PCR was performed in accordance with standard procedures with 2 µM of each primer and 2U Takara Ex-Taq DNA polymerase. The reaction mixture was subjected to an initial denaturation at 95°C for 3 min followed by 35 cycles of 95°C for 30 sec, 61°C for 30 sec, and 72°C for 2 min, and concluded with a final DNA extension at 72°C for 5 min.

**Figure 2.**
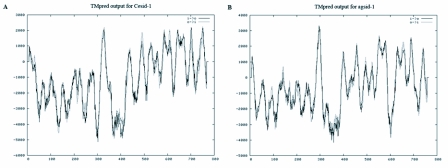
Prediction graphics of transmembrane topology of the SID-1 from *Caenorhabditis elegans* (A) and *Aphis gossypii* (B). The peaks over zero show transmembrane domains of the protein. This diagram shows that both proteins have a long N terminus that is followed by 11 transmembrane domains assembled in similar patterns.

### Analysis of sid-1-like gene sequence with on-line tools

The DNA sequences were analyzed with DNAstar software. Blast research was performed at http://www.ncbi.nlm.nih.gov/blast/. Sequences were aligned with ClustalX software and viewed with GeneDoc software (Thompson et al. 1997). Protein signal peptide was predicted with SignalP server on-line tools, http://www.cbs.dtu.dk/services/SignalP/. Transmembrane protein topological structure was analysized with TMpred on-line tools, http://www.ch.embnet.org/software/TMPRED_form.html. The amino acid sequences of sid-1-like transcripts from different species used for construction of a phylogenetic tree were downloaded from GenBank. Homologic comparisons were made using ClustalX software (Thompson et al. 1997). Phylogenetic relationships were calculated using SEQBOOT, PROTDIST and NEIGHBOR programs in Phylip software. The phylogenetic tree was created using TreeView software (Felsenstein. 1993).

## Results and Discussion

### Sid-1-like transcripts cloned from aphids

Different combinations of 5 degenerate PCR primers were all used to clone the fragments of sid-1-like genes from the two aphid species. The target bands were cloned with the primer pair of sid-i up 3 and sid-1 down 2. The fragment cloned from *A*. *gossypii* was 701 bp in length and that from *S*. *avenae* was 683 bp. They shared 90% similarity in nucleotide sequence and 96% in amino acid sequence. One of them was selected for cloning its full length with RACE procedure.

A full length transcript of cotton aphid sid-1-like gene (Ag-sid-1) was cloned, which contained 272 bp 5′ UTR, 494 bp 3′ UTR, and 2301 bp ORF encoding 766 amino acids. The predicted protein (Ag-SID-1) was 88 KDa and had a signal peptide of 17 amino acids at its N-terminus. This transcript shared highest similarity with the reported sid-1-like transcript in *S. americana* (53%), followed by those of *A.mellifera* (44%), *T.castaneum* (32–44%), *B.mori* (38–42%) and *C*.*elegans* (25%) (see Figure 1). Furthermore, according to the analysis with TMpred on-line tools, the topological structure of Ag-SID-1 is very similar to the well studied SID-1 from *C. elegans* (Figure 2), for they both have the same numbers of predicted membrane-spanning regions and a long N-terminus that also assemble in similar pattern. Thus, it is reasonable to conclude that the cloned transcript is the sid-1-like gene in aphid.

### Phylogenetic relations of sid-1-like genes from various organisms

**Figure 3.**
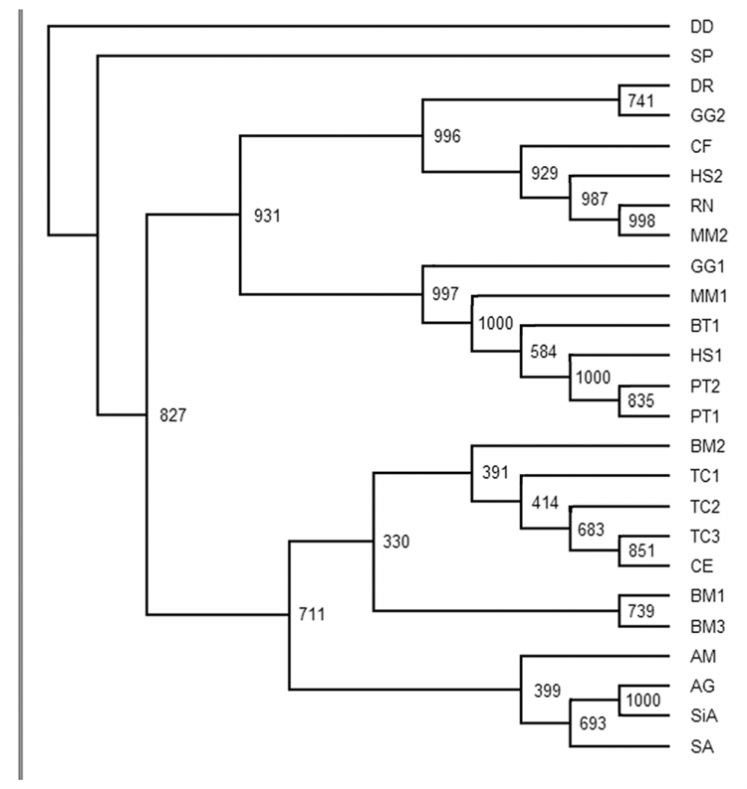
Phylogenetic tree based on amino acid sequences of sid-1-like genes available: DD, *Dictyostelium discoideum*, GenBank Accession No. XM630477. SP, *Strongylocentrotus purpuratus*, GenBank Accession No. XP789210. DR, *Danio rerio*, GenBank Accession No. XM694078. GG2, *Gallus gallus*, GenBank Accession No. XM001233565. GG1, *Gallus gallus*, GenBank Accession No. XM416544. CF, *Canis familiaris*, GenBank Accession No. XM540509. HS1, *Homo sapiens*, GenBank Accession No. NM017699. HS2, *Homo sapiens*, GenBank Accession No. NM001040455. RN, *Rattus norvegicus*, GenBank Accession No. XM001068825. MM1, *Mus musculus*, GenBank Accession No. NM198034. MM2, *Mus musculus*, GenBank Accession No. NM172257. BT, *Bos taurus*, GenBank Accession No. XM585013. PT1, *Pan troglodytes*, GenBank Accession No. XM001158341. PT2, *Pan troglodytes*, GenBank Accession No. XM526266. BM1, BM2, BM3, *Bombyx mori*, cited from George and Gene (2006). TC1, *Tribolium castaneum*, GenBank Accession No. XM969743. TC2, *Tribolium castaneum*, cited from George and Gene (2006). TC3, *Tribolium castaneum*, GenBank Accession No. XM969161. CE, *Caenorhabditis elegans*, GenBank Accession No. AF478687. AM, *Apis mellifera*, GenBank Accession No. XM395167. AG, *Aphis gosspyii*, GenBank Accession No. EF533711. SiA, *Sitobion avenae*, GenBank Accession No. EF533713. SA, *Schistocerca americana*, GenBank Accession No. AY879097

A phylogenetic tree was generated using 25 amino acid sequences of sid-1-like genes from 16 species (Figure 3). When the sid-1 homologous sequence from *D. discoideum* was used as an outgroup, the phylogenetic tree branched into 3 groups: echinus, nematodes and insects, and vertebrates. Within nematode and insect group, the sid-1-like transcripts from nematodes, beetles and moths assembled into one subgroup, and those from aphids, locusts and bees into the other. There were also two subgroups in the vertebrate group. But the sid-1-like transcripts from the same mammal frequently assembled into different subgroups. Because of the small amount of data available, it is difficult to analyze the evolution and duplication of sid-1-like genes in different animals, Three groups branching from the phylogenetic tree implies that sid-1-like genes have evolved from bacteria, echinus, nematode, insect to vertebrate, On the other hand, sid-1-like genes in different species shared low similarity, This suggests that sid-1-like genes have had a long independent evolutional history, Thus, it is reasonable to deduce that sid-1-like genes might evolve from an ancient gene, and might distribute in wide range of organisms (Table 1).

**Table 1.**
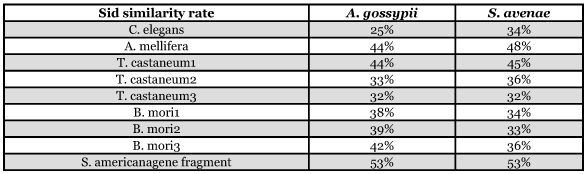
Similarity analysis of conserved gene regions among aphid sid-1 with other insect species and C.elegans

Sid-1 has been proved to be necessary for systemic RNAi which functions as a defense against viruses and in the control of gene expression (Winston et al, 2002), However, sid-1-like genes are not found in all organisms, They may have been lost randomly during evolution, and may have other compensatory dsRNA pathways that fulfill their important functions, Previously, the insect without sid-1-like genes were thought to have only cell-autonomous RNAi, Recently, the pea aphid was found to have systemic RNAi (Mutti et al. 2006), But a sid-1-like gene was not found in the recently sequenced pea aphid genome using bioinformatics (unpublished observations), This implies that the sid-1-like gene may be not necessary for systemic RNAi and the pea aphid may have other compensatory dsRNA pathways, which obviously needs further study.

## **Abbreviations**

dsRNA -: double stranded RNA,
ORF -: open reading frame,
RNAi -: RNA interference,
sid -: systemic interference defective,
siRNA -: small interfering RNA,
UTR -: untranslated region,
RACE -: rapid amplification of cDNA ends
